# Nursing leadership in intensive care units and its relationship to the work environment^1^

**DOI:** 10.1590/0104-1169.0150.2531

**Published:** 2015

**Authors:** Alexandre Pazetto Balsanelli, Isabel Cristina Kowal Olm Cunha

**Affiliations:** 2 Doctoral student, Escola Paulista de Enfermagem, Universidade Federal de São Paulo, São Paulo, SP, Brazil. RN, Escola Paulista de Enfermagem, Universidade Federal de São Paulo, São Paulo, SP, Brazil. Scholarship holder from Coordenação de Aperfeiçoamento de Pessoal de Nível Superior (CAPES), Brazil; 3 PhD, Associate Professor, Escola Paulista de Enfermagem, Universidade Federal de São Paulo, São Paulo, SP, Brazil

**Keywords:** Nursing, Leadership, Intensive Care Units, Health Facility Environment

## Abstract

**AIM:**

To establish whether there is any relationship between the work environment and nursing leadership at intensive care units (ICUs).

**METHOD:**

Correlational study conducted at four ICUs in southern São Paulo (SP), Brazil. The study population was comprised of 66 pairs (nurses and nursing technicians) established by lottery. The nurses responded to three instruments: 1) characterization; 2) a validated Portuguese version of the Nursing Work Index Revised (B-NWI-R); and 3) Grid & Leadership in Nursing: ideal behavior. The nursing technicians responded to 1) characterization and to 2) Grid and Leadership in Nursing: actual behavior, relative to the corresponding randomly-assigned nurse. The data were analyzed by means of analysis of variance (ANOVA) at p ≤ 0.05.

**RESULTS:**

The work environment was not associated with actual nursing leadership (p = 0.852). The public or private nature of the institutions where the investigated ICUs were located had no significant effect on leadership (p = 0.437). Only the nurse-physician relationship domain stood out (p = 0.001).

**CONCLUSION:**

The choice of leadership styles by nurses should match the ICU characteristics. Leadership skills could be developed, and the work environment did not exert any influence on the investigated population.

## Introduction

The labor market has been increasingly demanding active leadership from nurses. To meet this demand, understanding the relationship between this variable and other variables is critical for the formulation of action plans that promote the development of this skill.

Concern with nursing leadership is crucial in intensive care units (ICUs) due to their dynamic nature, which results from the severe condition of the admitted patients and which requires nurses to provide highly complex care. Within this setting, nurses must lead their staff to achieve the best outcomes, for which leadership skills are essential. Thus, the relationship between that dynamic, interactive environment and nursing leadership represents an appropriate subject of study. 

The correlation between leadership and the work environment has been studied by several authors at the institutional level but not specifically in the ICU setting. A total of 24 studies included in a systematic review^(^ 1 ^)^ showed that leadership styles focusing on people and relationships contributed to improving the outcomes of the nursing workforce, the work environment, and the productivity and effectiveness of health organizations. By contrast, the staff satisfaction was lower in 10 studies in which leadership focused on tasks. 

Transformational leadership increased the nurses' satisfaction in all institutional areas, reduced burnout, and provided a favorable work environment^(^ 2 ^)^.

Another study^(^ 3 ^)^ designed a test model in which the authentic leadership of managers was combined with the nurses' perception regarding structural empowerment, *performance*, and personal satisfaction. The sample consisted of 280 nurses who responded to instruments for data collection on each of the assessed variables. Leadership had a significant and positive influence on all the investigated attributes. 

A study that investigated the role of organizational power and the leader's personal influence in the creation of a high-quality professional practice environment for nurses^(^ 4 ^)^ found a direct and positive relationship.

To summarize, leadership impacts the work environment. However, the measure in which the environment might interfere with the development of leadership is still a gap that deserves investigation, especially in intensive care units because no studies assessing that relationship in this particular setting could be located. 

Thus, the present study poses the following research question: Is there a relationship between the ICU work environment and nursing leadership? The answer to this question will help advance the knowledge on this subject and support nursing management, modifying the development of that skill based on the results. In addition, the critical nature of the ICU setting, the use of high technology, and the presence of interdisciplinary staffs further stimulate this type of inquiry. Thus, the aim of the present study is to investigate whether there is any relationship between the ICU work environment and nursing leadership. 

## Method

The present correlational study involved a systematic investigation of the nature of the relationship or association between variables^(^ 5 ^)^. The study was conducted at four ICUs in the southern area of São Paulo County, SP, Brazil. Those ICUs were located at tertiary hospitals and provided general intensive care to adults with clinical or surgical problems. Two ICUs belonged to private institutions (A and B) and two to public teaching hospitals (C and D). The reason for that choice was to compare public and private settings as well as for the investigators' convenience. 

ICUs at public and private institutions were selected based on the assumption that from the macromanagement perspective, they exhibited differences. That is, the management of the physical, material, and human resources, quality, and safety was different. This assumption was based on the investigators' professional work experience in both settings. 

The study population consisted of registered nurses and nursing technicians with at least six months of work experience in an ICU. This criterion was established to ensure that the participants had the minimum experience in intensive care required for a sound assessment. 

The procedure for data collection started with the principal investigator's making contact with the nurses to inform them of the aims of the study. Next, the nurses randomly selected one of the nursing technicians on staff, who was also consulted, by lottery. After agreeing to participate, all of the volunteers signed an informed consent form. 

The nurses were blind as to the nursing technicians they had selected, as the latter were identified by numbers that were only known to the principal investigator. By contrast, the nursing technicians were aware of the identity of the leader they had to evaluate, as his or her name was included in the instrument for data collection. Anonymity was ensured to avoid any influence that could interfere with the subjects' responses. 

An envelope containing the following three instruments for data collection was delivered to the nurses: 1) characterization: comprising data on age, gender, time since graduation, job at the institution and in the ICU, attendance of graduate courses, and acquaintance with the topic of leadership. Those variables were selected based on the investigators' experience and the intention to assess their relationship to leadership; 2) a validated Portuguese version of the *Nursing Work Index Revised* (B-NWI-R)^(^ 6 ^)^; and 3) Grid & Leadership in Nursing: ideal behavior^(^ 7 ^)^.

The selected nursing technicians were given an envelope that contained the following: 1) the aforementioned instrument for characterization; and 2) Grid & Leadership in Nursing: actual behavior^(^ 7 ^)^ to complete relative to the corresponding randomly-assigned nurse. 

As a result, pairs were formed between a nurse and the nursing technician randomly selected by him or her to evaluate the former's perception on ideal leadership behavior and the assessment made by the latter on the actual performance of the nurse as his or her immediate leader. 

The participants responded to the instruments away from the work environment. A date was scheduled to hand the questionnaire directly to the principal investigator. The principal investigator did not hold a leadership position at any of the investigated ICUs. 

The characteristics of each instrument are described in the following. The *B-NWI-R* ^(^ 6 *)* is based on the Nursing Work Index (NWI), which was drafted in 1989. It consists of 65 items. The *Nursing Work Index Revised* (NWI-R)^(^ 8 ^)^ was formulated to make the NWI shorter and also to measure particular characteristics of the work environment that are favorable to nursing professional practice. 

The NWI-R consists of 57 items, 15 of which were conceptually distributed across three subscales: autonomy, control over the practice setting, and the nurse-physician relationship. A total of 10 of these 15 items were clustered to form a fourth subscale: organizational support^(^ 8 ^)^.

Conceptually, the subscales are defined as follows^(^ 8 ^)^: autonomy (five items) and control (seven items) represent the freedom nurses have to solve problems that affect the quality of nursing care; the nurse-physician relationship (three items) concerns the professional respect needed for effective communication regarding shared goals in patient care; organizational support (10 items derived from the three aforementioned subscales) concerns the situations in which the organization provides the necessary support for nursing professional practice. 

The measurement scales are 4-point Likert scales; the lower the score, the greater the presence of attributes favorable to professional nursing practice. The score of each subscale is calculated based on the average scores of the responses given by subjects and ranges from one to four^(^ 8 ^)^.

Because the NWI-R was translated and adapted for the Brazilian population^(^ 9 ^)^ and the subscales were validated (B-NWI-R)^(^ 6 ^)^, they were used in the present study.

*Grid & Leadership in Nursing: ideal and actual behavior* ^(^ 7 ^)^ consists of two instruments based on the grid theory to assess the ideal leadership behavior of nurses and the actual views of staff members in this regard. One instrument is thus responded to by the staff leader, and the other by a staff member. As both instruments are appropriate for the Brazilian setting and were subjected to apparent and content validation^(^ 7 ^)^, they are used in the present study.

The instrument consists of 25 statements with four answer options, attributed different scores and categorized as follows: entirely desirable (four points), desirable (three points), undesirable (two points), and entirely undesirable (one point)^(^ 7 ^)^.

Each statement concerns one style of leadership (1.1; 1.9; 5.5; 9.1; and 9.9)^(^ 7 ^)^. The leadership style attributed the highest score represents how a nurse exerts leadership as a function of his or her conception of ideal behavior and the view of a member of his or her staff regarding the actual situation^(^ 7 ^)^.

The data were collected from October 2012 to March 2013. The study was approved by the research ethics committee (Comitê de Ética em Pesquisa - CEP) of the Federal University of São Paulo (Universidade Federal de São Paulo - UNIFESP) under protocol number 0839/10.

Before the onset of data collection and following the CEP approval, a pretest was performed with a random sample comprising 13 nurses and 13 nursing technicians who did not participate in the full study. No difficulty in the interpretation of the questionnaires used was reported. 

The data on characterization were analyzed by means of descriptive statistics. Analysis of variance (ANOVA) with a p-value equal to or lower than 0.05 was used to investigate the correlation between the work environment and actual nursing leadership, which were the variables of interest in the present study. 

## Results

The response rate was 54.5%, i.e., 121 nurses and nursing technicians returned the questionnaires. However, the sample consisted of 66 nurse-nurse technician pairs, distributed as follows: ICU A = 34, ICU B = 3, ICU C = 16, and ICU D = 13.

Of the 66 nurses who were interviewed, 48 (72.7%) were female; their shift distribution was as follows: morning, 15 (22.7%); morning and afternoon, 5 (7.6%); afternoon, 11 (16.7%); and night, 35 (53%). The morning and afternoon shift was included because the work schedule at one of the institutions was 12x36 h during the day.

All of the nurses (100%) had been introduced to the subject of leadership during their undergraduate studies; 29 (43.99%) had attended lectures, and 28 (42.4%) had been given special training on the subject. A total of 60 nurses (90.9%) had attended specialized courses, most of which were on intensive care, 28 (46%); 10 among the latter had attended specialized courses in other fields. 

Of the group of nursing technicians, 41 (62.1%) were female, worked the same shifts as their superiors, and had been less exposed to the subject of leadership: 42 (63.6%) in a technical course, 18 in lectures (27.3%), 16 (24.3%) in training sessions, and 7 (10.6%) in other contexts, in addition to coursework for 11 (16.7%) who were studying nursing. 

The technicians were slightly older (average age: 34.7 years old) than the nurses (average age: 32.9 years old), and their time since graduation was longer (on average, 10 years). The length of work at the institution and ICU was longer among the nurses (on average, 6 and 5.2 years, respectively) compared to the nursing technicians (on average, 5.1 and 4.8 years, respectively). The fact that 35 pairs (53%) worked the night shift is deserving of particular attention. 

The average scores on the B-NWI-R were similar among the four investigated ICUs (mean = 1.95; 95% CI: 1.85-2.05): ICU A, 1.88 (95% CI: 1.74-2.02); ICU B, 2.29 (95% CI: 1.87-2.71); ICU C, 1.90 (95% CI: 1.69-2.10); and ICU D, 2.10 (95% CI: 1.83-2.37).

The internal consistency of the B-NWI-R domains was assessed by means of Cronbach's alpha, the values of which were: total B-NWI-R = 0.819; B-NWI-R autonomy = 0.645; B-NWI-R control over practice setting = 0.732; B-NWI-R nurse-physician relationship = 0.702; and B-NWI-R organizational support = 0.748.

Among the population of 66 responding nurses, 65 (98.5%) rated leadership style 9.9 as the ideal leadership style. However, when their actual leadership style was assessed by the nursing technicians, the results were as follows: 9.9 was attributed to 42 nurses (63.6%); 9.1 to eight (12.1%); 5.5 to 9 (13.6%); 1.9 to six (9.1%); and 1.1 to one (1.5%). Thus, 42 (63.6%) leaders were assessed in a satisfactory manner (95% CI [50.8%; 74.8%]).

Table 1 shows the intersection between the average scores on the B-NWI-R and the actual leadership styles of nurses independent of the particular ICUs. 

**Table 1 -:** Descriptive statistics corresponding to the total score on the B-NWI-R per actual leadership style. São Paulo, SP, Brazil, 2013.

Actual leadership styles	*Brazilian Nursing Work Index Revised* (B-NWI-R)						
	N	Minimum	Maximum	Mean	Standard deviation	95% CI*	
						Lower	Upper
	66	1.00	3.00	1.95	0.40	1.85	2.05
1.1	1	2.07	2.07	2.07	-	-	-
1.9	6	1.60	2.53	2.12	0.34	1.76	2.48
5.5	9	1.33	2.60	1.93	0.45	1.59	2.28
9.1	8	1.47	2.20	1.90	0.29	1.66	2.14
9.9	42	1.00	3.00	1.93	0.43	1.80	2.06

* 95% confidence interval

The data presented above show that there was no difference between the work environment and actual nursing leadership (p = 0.852). Styles 9.9 and 9.1 were associated with the lowest average scores on the B-NWI-R. In comparison, the means for styles 1.1 and 1.9 tended to be higher but not significantly. 

Actual leadership style was not significantly related to the various B-NWI-R domains, as shown in Table 2. 

**Table 2 -:** Descriptive statistics corresponding to the B-NWI-R domains per actual leadership style. São Paulo, SP, Brazil, 2013.

*Brazilian Nursing Work Index Revised* (B-NWI-R) domain	Group	N	Minimum	Maximum	Mean	Standard deviation	95% CI*		p
							Lower	Upper	
Autonomy	All	66	1	3.4	1.92	0.49	1.79	2.04	
	1.1	1	2	2	2	-	-	-	
	1.9	6	1.6	2.4	2.03	0.32	1.7	2.37	0.977
	5.5	9	1	3.2	1.87	0.7	1.33	2.41	
	9.1	8	1.2	2.4	1.9	0.41	1.55	2.25	
	9.9	42	1	3.4	1.91	0.49	1.76	2.06	
Control over practice setting	All	66	1	3.57	2.01	0.51	1.88	2.13	
	1.1	1	2	2	2	-	-	-	
	1.9	6	1.71	2.86	2.24	0.55	1.66	2.81	0.847
	5.5	9	1.57	2.71	1.97	0.36	1.69	2.25	
	9.1	8	1.43	2.57	1.95	0.41	1.6	2.29	
	9.9	42	1	3.57	2	0.55	1.82	2.17	
Nurse-physician relationship	All	66	1	3	1.85	0.47	1.74	1.97	
	1.1	1	2.33	2.33	2.33	-	-	-	
	1.9	6	1	3	2	0.76	1.2	2.8	0.632
	5.5	9	1	2.67	1.96	0.51	1.57	2.36	
	9.1	8	1	2.67	1.79	0.56	1.32	2.26	
	9.9	42	1	2.67	1.81	0.4	1.69	1.93	
Organizational support	All	66	1	3.1	1.96	0.43	1.86	2.07	
	1.1	1	2.2	2.2	2.2	-	-	-	
	1.9	6	1.8	2.6	2.22	0.37	1.83	2.61	0.539
	5.5	9	1.4	2.7	1.97	0.45	1.62	2.31	
	9.1	8	1.4	2.1	1.84	0.28	1.61	2.07	
	9.9	42	1	3.1	1.95	0.46	1.8	2.09	

* 95% confidence interval

As the number of pairs was heterogeneous among the investigated ICUs and to test for differences in the B-NWI-R scores and the actual leadership styles between the nurses at the private (ICU A and B) and public (ICU C and D) hospitals, the corresponding ICUs were pooled for comparison. 

The average total score on the B-NWI-R was 1.91 (95% CI: 1.83-2.15) in the ICUs at the private hospitals and 1.99 (95% CI: 1.78-2.05) in the ICUs at the public hospitals, p = 0.459. The correlation of those scores with the actual nursing leadership style exhibited p = 0.437. 

The average score for each B-NWI-R domain was analyzed relative to the actual leadership style of the nurses at the public and private hospitals. The results were as follows: autonomy, p = 0.629; control over practice setting, p = 0.676; nurse-physician relationship, p = 0.001; and organizational support, p = 0.254. 

**Figure 1 -:**
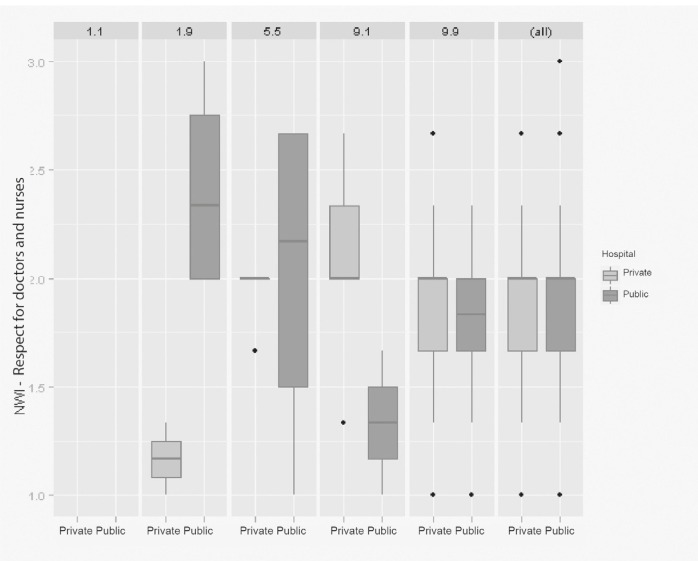
Boxplot representing the nurse-physician relationship domain per actual leadership style and hospital type. São Paulo (SP), Brazil, 2013.

The ANOVA showed that the effect of the interaction between hospital type and actual nursing leadership style was significant only in the nurse-physician relationship domain (p = 0.001), as shown in Figure 1.

The scores on the B-NWI-R nurse-physician relationship domain relative to leadership style 9.1 were lower at the public hospital ICUs. The scores on the B-NWI-R relative to leadership style 1.9 were lower at the private hospital ICUs. Thus, the most favorable^(^ 6 ^)^ correlation between the leadership style in which the professional is most involved (9.1)^(^ 7 ^)^ and the mutual respect between nurses and doctors corresponded to the public hospital ICUs. 

## Discussion

The interest in studies assessing the impact of the work environment on the outcomes of care and management arose in the 1980s, when the American Academy of Nursing conducted a nationwide study to establish which characteristics of the work environment favored nursing professional practice. The institutions known as *magnet* hospitals shared the following features: management, professional practice, and professional development^(^ 8 ^)^.

Those attributes are assessed by the NWI-R^(^ 8 ^)^, which is the basis for B-NWI-R^(^ 6 ^)^ and which is anchored in the Nursing Professional Practice Model^(^ 10 ^)^. The latter is defined as a system (structure, process, and values) that lends support to nurses to control the care provided to patients and the setting in which care is provided. 

On those grounds, the results of the present study show that the work environment at the investigated ICUs is favorable for daily nursing practice (mean = 1.95; 95% CI: 1.85-2.05), regardless of the hospital being analyzed. 

One might thus infer that the ICU work environment is attractive for nursing professionals. This claim is corroborated by a study conducted in South Korea^(^ 11 ^)^ that investigated nurses' perceptions of the work conditions at the hospital and the ICU simultaneously by means of the NWI-R subscales. The sample consisted of 817 nurses from 39 ICUs at 15 hospitals. Based on the results of the multiple regression analysis, the work environment at the investigated hospitals was rated good, moderate, or poor as follows: good, two (13.4%); moderate, 10 (66.6%); and poor, three (20%). The corresponding assessment of the ICUs was as follows: good, nine (23.1%); moderate, 24 (61.5%); and poor, six (15.4%). 

The average score of 17 rural ICUs in the state of São Paulo (SP), Brazil^(^ 12 ^)^, on the B-NWI-R was 2.13, which was higher than the score found in the present study. Thus, the work environment at the four investigated ICUs was found to be healthy. 

However, no significant association was found between the average score on the B-NWI-R and the actual nursing leadership styles (p = 0.852). The same held true for the scores on the B-NWI-R subscales. 

Thus, the work environment was not found to influence the nursing leadership styles in the present study. However, only four of the B-NWI-R domains were validated for use in Brazil^(^ 6 ^)^. Once validated, the remaining domains might allow for a broader assessment of the work environment characteristics than the domains available at the present time. 

When the ICUs at the public and private hospitals were analyzed separately, no significant difference was found in relationship between the average B-NWI-R score and the nurses' actual leadership styles (p = 0.437). Only the nurse-physician relationship domain exhibited p = 0.001. It should be noted that the ICUs at the public hospitals exhibited the most favorable correlation between the leadership styles in which the professional is most involved, 9.1^(^ 7 ^)^, and the nurse-physician relationship domain. This finding leads us to reflect on how professional relationships occur, taking into consideration the fact that as teaching hospitals, the public hospitals might have been more favored. Nevertheless, experience in the ICU allows the inference that interdisciplinary interactions do occur and that they are generally healthy, given the severity of the patients under staff care. 

Leadership has been associated with several variables, including patient satisfaction. There is strong evidence^(^ 12 ^)^ of an association between positive leadership behaviors and increased patient satisfaction and reduced adverse events^(^ 12 ^)^.

In one study^(^ 13 ^)^, 29% of job satisfaction, 22% of organizational commitment, and 9% of productivity were explained by the use of leadership behavior. Those findings are in accord with the results of three studies^(^ 14 ^-^ 16 ^)^ in which the variables of interest were job satisfaction, organizational climate^(^ 14 ^-^ 15 ^)^, and *burnout* ^(^ 16 ^)^. Leadership was found to exert a positive, direct influence on those indicators^(^ 14 ^-^ 16 ^)^. 

Based on those findings and the fact that the work environment might attract and retain excellent professionals, according to the Nursing Professional Practice Model^(^ 10 ^)^, it was hypothesized that such strong characteristics might influence nursing leadership styles, given the lack of studies in the literature that seek to answer this question. 

The results of the present study show that the work environment is similar at the four investigated ICUs. No relationship is found between the work environment and nursing leadership in the assessed population. These findings give rise to several questions that might be answered by future studies: Are ICUs different from other hospital areas? Would the application of the entire NWI-R, following its validation for the Brazilian context, allow for a better assessment of work environments and subsequent replication of the method used in the present study? Would the inclusion of additional ICUs allow for a more comprehensive analysis of this problem within the intensive care setting?

## Conclusion

The work environment was not associated with actual nursing leadership (p = 0.825), and the same held true in the case of the B-NWI-R subscales. The public or private nature of the hospitals where the investigated ICUs were located did not exhibit a direct relationship with leadership (p = 0.437). Only the nurse-physician relationship domain stood out, p = 0.001. 

The present study had the following limitations: sample size and convenience sampling; application of only four of the B-NWI-R domains; few studies that used the NWI-R in the intensive care setting; and the fact that the leadership framework used dates from the 1990s. 

Based on these results, nursing managers might conclude that nurses ought to develop leadership skills and that the work environment does not exert influence on the study population. Thus, nurses require individual development plans that include the types of knowledge, skills, attitudes, values, and deliverables needed to exert leadership. Within this context, the role of Nursing Training Centers also stands out because they are tasked with the training and development of leaders fit for work in the intensive care setting, using the findings of the present study. 

Because the work environment was not associated with nursing leadership in the investigated ICUs, further scientific studies with larger samples might be conducted to confirm this result and to identify other variables the may interfere with nursing leadership in the intensive care setting. 
